# Regionalization of the Developing Hypothalamus: The Prosomeric and Tripartite Models

**DOI:** 10.3390/cells15121085

**Published:** 2026-06-15

**Authors:** Marika Kapsimali

**Affiliations:** Institut of Biology of the Ecole Normale Superieure (IBENS), INSERM U1024, 75005 Paris, France; marika.kapsimali@inserm.fr

**Keywords:** retromammillary hypothalamus, gene regulatory networks, scRNAseq, forebrain, mammillary hypothalamus

## Abstract

The hypothalamus is a conserved structure in the brain of vertebrates that plays key roles in the organism’s homeostasis through different functions, including regulation of temperature, sleep, energy balance and reproduction. The hypothalamus is composed of juxtaposed nuclei, showing dense organization. During development, anterior, tuberal and posterior subdivisions are observed. However, because of the complexity of its organization, the development of hypothalamic architecture is the subject of different developmental models. First, the prosomeric model argues that the hypothalamus is an anterior structure, divided into two distinct hypothalamic prosomeres, and proposes subdivisions within them based on gene expression patterns. Second, the recently established tripartite model based on gene expression patterns, scRNAseq, fate mapping, and functional assays proposes that the hypothalamus is largely a diencephalic structure, organized in areas/nuclei, and the retromammillary area is a posterior hypothalamic boundary within the diencephalon, ventral to the ZLI. In addition to the prosomeric and tripartite models, key molecular processes that underlie the formation of hypothalamic areas are discussed. Finally, this work argues for the tripartite model as the key model of hypothalamic formation in vertebrate species.

## 1. Introduction

The hypothalamus is one of the most ancient structures in the brain of vertebrates. It maintains energy balance, regulates temperature and sleep, controls growth, enables reproduction and parenting behavior, and establishes circadian rhythms. Anatomically, the hypothalamus is divided along the antero-posterior (A-P) axis into the anterior hypothalamus, tuberal hypothalamus and mammillary hypothalamus. Each one of these hypothalamic areas is further organized into specific nuclei which contain an extraordinary diversity of neuronal and glial types [1,2]. In particular, in addition to glutamate, GABA or dopamine, hypothalamic neurons synthetize one or more of the following peptides and receptors: Agrp, Avp, Avpr1a, Cck, Crh, Dynorphin, Enk, Esr1, Gal, Ghrh, Glp1r, Gnrh, Grp, Grpr, Hcrt, Kiss1, Kiss1r, Lepr, MCH, neurokinin B, Nms, NPY, Nts, Oxt, POMC, Pmch, Pnoc, PR, Rprm, Prok2, Sst, Tac1, Trh, Ucn3, Vip, Vipr2. Research during the last 30 years has revealed many functions of these peptides into intra-hypothalamic or hypothalamic-extra-hypothalamic neuronal circuits [1,3,4].

Despite the importance of the hypothalamus in the life of vertebrate species, the regionalization of the developing hypothalamus has been unclear until recently. The aim of this review is to discuss recent progress in our comprehension of the regionalization of the developing hypothalamus, in particular by taking into account the prosomeric [5] and tripartite hypothalamic subdivisions [6].

## 2. Developing Hypothalamic Progenitor Domains Define Adult Nuclei

**Figure 1:**
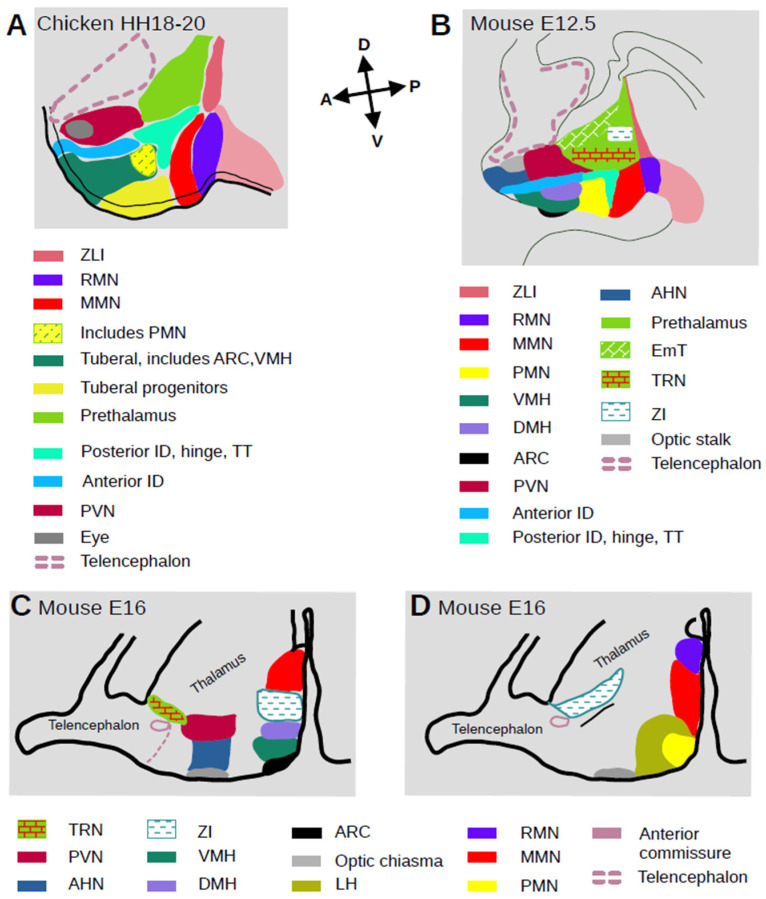
Color-coded cartoons showing the regional subdivisions in the developing hypothalamus. (**A**): chicken (HH18-20), (**B**): mouse (E12.5). Arrows indicate A-P and D-V axes. Adapted from [6,14]. (**C**,**D**): cartoons of sagittal sections of a mouse E16 brain showing related positions of hypothalamic and prethalamic nuclei. Section (**D**) is lateral to (**C**). Adapted from [7].

In mammals, the preoptic area has been debated as a telencephalic rather than a diencephalic region [5], and it is analyzed apart from the hypothalamus. Therefore, it is considered that the anterior hypothalamus is composed of the anterior hypothalamic (AHN), supraoptic (SON), suprachiasmatic (SCN), and paraventricular (PVN) nuclei. The tuberal hypothalamus contains the lateral hypothalamic area (LH), arcuate (ARC), dorsomedial (DMH), and the ventromedial (VMH), and its migrated part, tuberal (TuN) hypothalamic nuclei. The more posterior or mammillary hypothalamus includes the premammillary (PMN), mammillary (MMN), supramammillary or retromammillary (RMN) and posterior hypothalamic (PHN) nuclei (Figure 1A,B). The median eminence and pituitary stalk lie ventral to the tuberal hypothalamus. It is also important for this review to remind readers that the prethalamus (PTh) includes the thalamic eminentia (EmT), zona incerta (ZI) and thalamic reticular nucleus (TRN, [7] Figure 1A–D and Table 1 for abbreviations).

Single-cell RNA sequencing (scRNAseq) and gene regulatory networks (GRN) studies show that these adult anatomical subdivisions are already predicted by distinct progenitor subsets at embryonic stages [8,9]. These studies suggest how progenitors progress to differentiated neurons and provide a more accurate way of defining neurons than simply on the basis of their neuropeptide/neurotransmitter expression [9,10,11,12]. Each of the major subdivisions of the developing hypothalamus is identified at the peak of neurogenesis (E11–E13) along the A-P axis: postmitotic neuronal precursor cells of the paraventricular nucleus/supraoptic nucleus (PVN/SON), intrahypothalamic diagonal (ID) and tuberomammillary terminal (TT), ventromedial hypothalamus (VMH), arcuate nucleus (ARC), premammillary nucleus (PMN), mammillary nucleus (MMN), and retromammillary nucleus (RMN) (Figure 1A,B). The intrahypothalamic diagonal (ID) is parallel to the hypothalamus A-P axis and extends anterior until the optic recess. The tuberomamillary terminal (TT) is the border between mammillary and premammillary neuroepithelium (Figure 1A,B).

## 3. Induction and Regionalization of the Developing Hypothalamus

The hypothalamus (expressing *Foxd1*) is surrounded antero-dorsal by the telenephalon (expressing *Foxg1*), postero-dorsal by the prethalamus (expressing *Pax6*) and postero-ventral by the floorplate and diencephalic tegmentum (expressing *Arx*, *Fox1/2*, *Shh*) [8,9,11,13,14]. The hypothalamus originates from the hypothalamic floorplate (HypFP) cells, also known as rostral diencephalic ventral midline cells. In mouse, chicken and zebrafish neural plate stages, HypFP cells express *Shh*, *Bmp7* and *Nkx2.1* and lie anterior to the diencephalic floorplate cells which express *Foxa2* [15,16,17,18,19,20]. HypFP cells are induced through the combined action of Nodal, SHH and BMP signals from prechordal mesendoderm [15,16,17,18,21,22].

Nodal from the prechordal mesoderm triggers hypothalamic induction and patterning [21,23,24]. Abrogation of Nodal signaling in the zebrafish mutant Nodal-related 2 ligand (*cyc-/-*) or teratocarcinoma-derived growth factor 1 Nodal co-receptor (*oep-/-*) results in complete loss of hypothalamic tissue, consistent with cyclopia in these mutants [20]. Nodal signaling is required for the expression of both *Nkx2.1a* and *Nk2.1b* paralogs in the zebrafish hypothalamus.

In cultured chicken Hamburger–Hamilton stage 4 (HH4) explants, most cells express *Pax6*, but no *Nkx2.1*, while in HH6 explants, *Nkx2.1* expressing cells are present, some co-expressing *Pax6* [13]. At HH8, prethalamic and hypothalamic scRNAseq and RNA velocity show a trajectory from prethalamic-like to HypFP clusters [13,25]. From HH8 through HH20/21, there are progressively fewer prethalamic-like progenitors (expressing *Pax6*) and more hypothalamic cells (expressing *Shh/Nkx2.1*). Altogether, these results show that prethalamic cells are induced to hypothalamic fate. Therefore, they support the point that prethalamic and hypothalamic fate are tightly linked and argue for the formation of an initially common domain.

scRNA-seq analysis shows that *Pax6* expressing prethalamic-like cells express FST, a potent Nodal antagonist at HH8–HH10. In chicken embryo and dissected prospective hypothalamus cultured explants at HH4, treatment with anti-FST increases the *Nkx2.1/Shh* expressing hypothalamic domain and reduces the prethalamic *Pax6* domain, relative to controls. Conversely, exposure to FST almost eliminates *Nkx2.1* expression, and the number of *Pax6*-positive cells is increased in the embryo or hypothalamic explant. Therefore, prethalamic-derived FST inhibits hypothalamic specification and constrains the size of the developing hypothalamus [13]. In situ hybridization and RNAseq analysis of *Nkx2-1CreER/CreER* mutants and heterozygous littermate controls shows that *Nkx2.1* expression is required for the formation of the mediobasal hypothalamus including ARC, VMH, PMN, and MMN, but not RMN. At mouse E12.5, *Nkx2.1CreER/CreER* mutants show that both the relative expression levels and the number of cells expressing hypothalamic genes is reduced, while the number of cells in the prethalamic cluster is increased with no substantial overall change in the portion of neural progenitors. RNA velocity analysis shows altered trajectories between mediobasal hypothalamus, mammillary hypothalamus and prethalamus. Therefore, *Nkx2.1* not only promotes and maintains the identity of ventral hypothalamic progenitors but also actively represses prethalamic gene expression [8]. These results point out the cellular continuity between the prethalamus and hypothalamus, suggesting their formation from a common territory (prosomere 3, p3, Figure 2A–C) and that the existence of a boundary between the developing prethalamus (p3) and hypothalamus is highly unlikely (Figure 1A–D). They also provide a hint for a distinct developmental pathway of RMN compared to the *Nkx2.1*-dependent mediobasal hypothalamus [6,8].

BMP signaling is active in the developing tuberal hypothalamus. Remarkably, phosphorylated Smad1/5/8, key effectors of BMP signaling, are detected immediately after HH8 in anterior HypFP cells and propagate to more posterior HypFP cells. As tuberal cells are generated, the zone of active BMP signaling and underlying tissues becomes relatively posterior while the anterior tuberal domain expands. The late-specified HypFP cells show more sustained activation of phosphorylated Smad1/5/8 and become posterior tuberal gliogenic progenitors [26]. In conclusion, hypothalamic neuroepithelium-intrinsic factors are sufficient to maintain tuberal hypothalamic regionalization and neurogenesis.

BMP upregulates *Tbx2* in tuberal progenitors and, as a consequence, *Shh* expression is downregulated in a cell-autonomous manner [17]. This likely occurs by *Shh*-induced abrogation of *Smo* expression in ventral tubero-mammillary precursors. Downregulation of SHH signaling first promotes further proliferation of ventral tubero-mammillary progenitors and, second, induces *Emx2* expression. When BMP signaling is abrogated by *Chordin*, hypothalamic *Tbx2* expression is lost but restored by *Wif* (Wnt-inhibitory factor) activation [17]. Complementary to this, simultaneous activation of Nodal signaling and inhibition of Wnt-beta catenin signaling is required within the ventral midline floorplate-like cells to form a regionalized hypothalamus along the A-P axis. In zebrafish, abrogation of *Axin1*, an intracellular Wnt/beta-catenin inhibitor, results in a smaller hypothalamus of posterior identity expressing *Emx2* [27,28]. Interestingly, *Wnt8b* expression flanks the RMN hypothalamus, suggesting that it may receive more sustained posteriorizing signaling than the tuberal and mammillary hypothalamus and thus has a different fate [6].

SHH derived from the ventral forebrain neuroepithelium is essential for the differentiation of the anterior and tuberal hypothalamus [14]. *Shh* is initially broadly expressed in ventral hypothalamic progenitors and then rapidly downregulated in the ventral midline at the level of the tuberal hypothalamus [17,29]. When *Shh* is selectively removed from the hypothalamic basal plate and basal telencephalon in *Nkx2.1-CRE* × *ShhloxP/loxP* mice, *Foxb1*, *Rx*, *Sim1* and *Irx5* expression is maintained, showing that hypothalamic mammillary and retromammillary areas are present, respectively. However, hypothalamic cells are devoid of *Pomc* and *Nkx6.2* expression, showing that antero-tuberal hypothalamic nuclei are absent; *Nr5a1*, showing that differentiation of VMH nucleus is compromised; and *Lhx1*, *Lhx9*, *Lhx6* showing that the ID and tuberomamillary terminal are undifferentiated [30,31]. *Lef1* expression is decreased, suggesting that the premammillary neuroepithelium is reduced. As telencephalic *Foxg1* expression is not expanded, altogether these results suggest that ventral diencephalic cells still retain their hypothalamic identity but fail to undergo differentiation in *Nkx2.1-CRE* × *ShhloxP/loxP* mice [9,14]. In addition, these data highlight that there is no cell fate conversion between hypothalamic and telencephalic tissue, suggesting a possible boundary mechanism between the two regions, an argument against the prosomeric model.

In the E9.5 mouse hypothalamus, *Shh* is expressed in bilateral stripes adjacent to the ventral midline [32]. Neural progenitors immediately dorsal to the bilateral stripes of S*hh* are responsive to SHH signaling by *Gli1* expression. *Shh* expressing progenitors contribute to a subset of ARC neurons and ventrolateral, ventral and central neurons of the VMH. Descendants of *Gli1* expressing progenitors contribute to the DMH and dorsolateral VMH. Conditional loss of SHH signaling in the tuberal hypothalamus, by *Smo* deletion in *Gli1* expressing cells, results in a cell-autonomous reduction of proliferating progenitors of distinct DMH and VMH neurons. Furthermore, *ShhΔhyp* hypothalamus shows that SHH signaling is active after the dorso-ventral (D-V) identity is established. In particular, the non-dividing ventral midline cell population is laterally expanded, *Ascl1* expression and consequent neurogenesis are downregulated and *Pax6* expression, normally restricted to dorsal diencephalic areas (prethalamus), is ventrally expanded. As a result, tuberal hypothalamic nuclei are absent in *ShhΔhyp* embryos [33]. This is also a hint regarding the tight relation (fate conversion) between dorsal diencephalic (prethalamic) and hypothalamic cells as both derivatives of a common domain.

The anterior hypothalamic progenitor cells express *Rax/Rx/Rx3* [14]. When *Rx* is inactivated prior to E8.5 in *Rxf/f;CreER*-expressing [34,35] mouse embryos, the anterior and tuberal hypothalamus are devoid of *Shh* and the dorsomedial hypothalamus of *Otp*, *Pomc*, *Th1* and *Sst* expression. In a complementary manner, the ventromedial expression of *Fgf10*, *Otx2* and *Tbx3* expands dorso-medially [36]. *Rx-CreERT2:MADMGT/TG* tamoxifen-induced mice at E9.5 show at E11.5/E12.5 that the majority of *Rx* progenitors are multipotent and generate multiple neuronal subpopulations including glutamatergic VMH neurons and GABAergic TuN neurons [37,38]. Timed controlled inhibition of *S*HH signaling in zebrafish, at the hypothalamic cell differentiation stage, shows that *Shh* first induces and then downregulates *Rx3* expression. SHH-induced *Rx3* expression in proliferating progenitors is required for elongation of anterior/tuberal hypothalamus. Subsequent *Rx* downregulation is required for hypothalamic anisotropic growth, specification of *Shh* expressing (*Rx3* non expressing) cell fate in the anterior ventricular recess, and differentiation into *Pomc* (ARC), *Nr5a1a* (VMN) and *Th1*, *Otpb* tuberal/anterior cell fates [39].

RNA velocity on scRNAseq data infers that a hypothalamic differentiation trajectory is composed of *Six6/Fgf10*-expressing progenitor cells, *Six6/Ascl1/Isl1* expressing neurogenic precursors, and *Six6/Nr5aA1/Pomc* expressing neurons [13] In HH8-9 chicken, *Six6* and *Tbx2* are simultaneously expressed in anterior HypFP cells and resolve shortly after in anterior tuberal *Six6/Isl1* expressing cells, *Six6/Shh/Ascl1* expressing neurogenic progenitors, posterior tuberal *Tbx2* expressing progenitors, and *Foxa2/Shh* expressing HypFP cells [26]. In situ hybridization in E5 chicken shows that anterior, tuberal and mammillary domains are defined as *Six3*, *Six3* and *Fgf10*, and *Emx2* expressing progenitors, respectively. However, at 9–10 somite chicken, only tuberal *Six3* and *Fgf10* expressing progenitors are present. Anterior progenitors appear at 11 somites and mammillary progenitors even later at HH16. Despite the ~8-fold expansion of the entire hypothalamic region in the developing chick, the *Fgf10* expressing progenitor domain retains a constant size. *Fgf10* expressing progenitors are retained centrally and contribute to the tuberal domain in a cell autonomous manner. Furthermore, EdU and DiI/DiO injections show that anterior and mammillary progenitors develop from highly proliferating *Fgf10* expressing progenitors and begin to differentiate once displaced from the *Fgf10* expressing domain into anterior and mammillary domains [40,41]. In conclusion, the development of hypothalamic areas is heterochronous and characterized by anisotropic growth.

SHH signaling is involved in this anisotropic growth process of the hypothalamic areas relying upon *Fgf10*-expressing progenitors. At 9 somites, *Shh* and *Fgf10* are briefly co-expressed in the forebrain. From 11 somites, *Shh* becomes downregulated in *Fgf10* expressing progenitors [17] and strongly expressed in peripheral cells, including emerging anterior cells. Emerging anterior progenitors downregulate *Fgf10* and upregulate *Shh* and *p57Kip2*, then downregulate *Shh* as they differentiate. Compromised SHH signaling in controlled time periods results in the specific loss of anterior progenitor territory. Therefore, SHH signaling upregulation and subsequent downregulation results in progenitor proliferation from the pool of *Fgf10* expressing cells and anterior (but not tuberal) identity of those progenitors [41].

## 4. Transcription Factors Couple Regionalization and Differentiation of Hypothalamic Nuclei

Transcription factors (TFs) involved in early hypothalamic regionalization also regulate cell differentiation. To identify GRNs controlling hypothalamic regionalization and neurogenesis, Kim and colleagues apply SCENIC+ analysis in E11–E14 scRNA-seq and scATAC-seq datasets, which infers patterns of transcription factor (TF) activity [8,42]. Hypothalamic TFs, such as *Hmga1*, *Lin28a*, *Cited1*, and *Gbx2*, are active in early-stage neuronal precursor cells, whereas *Tcf4*, *Npas3*, *Nfia/b/x*, *Zbtb20* and *Tox3* are active later in neurogenesis. *Hmx2/3* and *Isl1* activate GABAergic gene expression across the tuberal, premammillary, and prethalamic regions. In addition, in *Neurog2* expressing neurons, GABAergic genes, e.g., *Slc32a1*, are activated by prethalamic/ID TFs (*Dlx1/2/5/6*, *Arx*, *Sp8*, *Meis2*) and repressed by glutamatergic TFs (e.g., *Barhl2*). Conversely, glutamatergic genes, e.g., *Slc17a6* are activated by both Neurog2 specific TFs (*Barhl1*, *Nhlh2*, *Nr4a2*, *Lmx1a*, *Neurod1*) and tuberal progenitor specific factors (*Nkx2–1*, *Sox14*). These glutamatergic genes are repressed by prethalamic specific TFs [8].

Loss of function experiments of distinct TFs are also available. The anterior hypothalamus is divided into two domains along the medio-lateral axis of the mantle zone. The median domain expresses *Sim1* and *Brn2* at E12.5 and generates PVN and SON, whereas the lateral expresses *Rgs4* and generates heterogeneous layer-organized neurons. Abrogation of *Sim1* activity in mice shows that *Sim1* is required for progenitor proliferation and activation of Brn2 expression [14,43,44].

*Otp* and *Dlx2* are expressed in TuN neurons and delineate TuN from *Nkx2.1* expressing VMH neurons [37]. In the progenitor domain of VMH, rare cells express *Otp* in a mutually exclusive manner with *Nr5a1* and migrate and accumulate in the presumptive TuN. Fate mapping analysis using *Nkx2.1-Cre:H2B-GFP* mice shows that nearly all *Otp* expressing and nearly all *Dlx2* expressing neurons in the TuN are GABAergic and derived from the *Nkx2.1* lineage [31,37]. Therefore, surprisingly, the progenitor domain of VMH is a common pool of progenitor cells, which gives rise to a glutamatergic VMH and a GABAergic TuN in a mosaic manner rather than as spatially segregated domains [37]. BrdU injection in *Dlx5/6-Cre;H2B-GFP* mouse embryos [45] shows that VMH-TuN neurons have a sequential and “outside-in” birth order, with early born neurons mainly located in lateral subdomains and late born neurons in medial subdomains. The VMHvl and TuN neurons are generated simultaneously, and VMH-TuN as a whole has a laminar-like structure [37]. In contrast to TuN, where rare cells co-express *Otp* and *DlxGFP*, in the LH area, either *Otp*- or *Dlx*-expressing neurons are found, and they are glutamatergic and GABAergic, respectively [37,46,47,48].

*Arx* is expressed in two linear domains: one that runs parallel to the basal domain of *Shh* expression toward the optic recess (parallel and part of ID) and the TT. This results in two domains with overlapping *Arx* and *Nkx2.1* expression but not *Shh* [14]. The expression of LIM homeodomain family members *Lhx1*, *Lhx8*, and *Lhx6* delineates the A-P axis of the ID/TT. LHX TFs define a series of nuclei residing both between and lateral to the PVN and VMH. In the anterior ID, at E12.5, *Lhx1* is expressed in the presumptive SCN. At E16.5, *Lhx1* is additionally expressed in a region immediately posterodorsal to the SCN [49]. The posterior domain of *Lhx1* expression overlaps with the anterior part of *Lhx8* expression in the ID. *Lhx8* is further extended posterior to *Lhx1* and reaches the limit of *Lhx6* expression. The *Lhx8* expression domain of the ID corresponds to the central region of the DMN. *Lhx6* is expressed in the ID until the posterior domain of *Arx* and *Nkx2.1* expression. *Lhx9* is expressed postero-ventral to *Lhx8* and immediately ventral to the *Lhx6*-positive zone of the ID in a *Nkx2.1*-negative ventral hypothalamic area [14]. Like *Lhx6*, *Lef1* is expressed in the ventral posterior ID and TT domains. *Lhx6CreER/+;Ai9* mice show that distinct *Lhx6* cells co-express distinct transcription factors: *Nkx2.1* in the ID, *Dlx1/2* in the TT, and *Nkx2.2* in the hinge between the posterior ID and dorsal TT. Abrogation of *Nkx2.1*, *Nkx2.2*, and *Dlx1/2* selectively eliminates hypothalamic *Lhx6* expression in the ID, hinge and TT domains, respectively [49,50].

Presumptive ARC, median eminence, pituitary stalk and posterior pituitary develop from a midline domain of the basal hypothalamus expressing *Tbx3* [29,51,52]. *Tbx3* is required for the differentiation of pro-opiomelanocortin (POMC) or neuropeptide Y (NPY) and agouti-related peptide (AgRP)-expressing neurons [37,52,53,54]. ARC neurons are divided in distinct POMC and NPY/AgRP and TH neurons by combinatorial *Tbx3*, *Otp*, and DlxGFP expression, respectively (*DLX5/6-Cre;H2B-GFP* [45]). scRNAseq data of adult mouse ARC neurons supports the compartmentalization of ARC in distinct cell subpopulations [55]. *Otp* and DlxGFP are expressed in ARC differentiating neurons in a mutually exclusive salt-and-pepper pattern [37]. Abrogation of *Otp* activity results in the absence of NPY, AgRP, and Sst expressing neurons and reduced *Gad 1*, GR and *Bsx* expression. In contrast, POMC and GHRH are expressed in *Otp-/-* mice, and the number of TH and *Dlx1* expressing neurons is similar to the wild-type. Therefore, in the developing ARC, *Otp* does not repress the expression of *Dlx1/2*. However, DLX1/2 directly bind and repress *Otp* expression as it is shown by ChIPseq analyses, luciferase or reporter expression controlled by *Dlx1/2* regulatory elements [56].

In the posterior hypothalamus, *Lef1* and *Irx5* are expressed in the PMN and RMN neuroepithelium, which lie immediately anterior and posterior to MMN neuroepithelium, respectively [14]. Neurogenic progenitor analysis by Scenic+ infers that *Neurog2* expressing cells activate early-stage RMN-specific factors such as *Lmx1a*, *Foxa1/2* and *Irx3/5*, while early-stage *Ascl1* expressing cells activate prethalamus specific TFs, including *Dlx1/2/6*, *Sp8/9*, and *Arx* [8]. These results suggest that the differentiation of RMN and prethalamic cells is independent of each other.

*Cck* is expressed in MMN and RMN neurons and activated by TFs from both MMN and RMN, such as *Nkx2.4* and *Uncx*. However, non-overlapping expression patterns of MMN and RMN-specific TFs are visible by E13.5, and MMN and RMN genes mutually antagonize their expression. For example, MMN-specific neuropeptides (*Pnoc*, *Npy*, *Nts*, *Cartpt*) are activated by MMN-specific TFs (*Lhx1/5*, *Uncx*, and *Nhlh2*) but directly repressed by RMN-specific TFs. In the RMN, *Tac1* is expressed in specific neuronal subpopulations but is absent from the MMN. GRN analysis infers that *Tac1* is directly activated by RMN-specific TFs (*Lmx1a/b*, *Foxa2*, *Barhl1/2*, and *Ebf2*) and repressed by the MMN specific factor *Nkx2.4*. These results reveal that early developmental pathways are common for MMN and RMN. However, TFs, which control differentiation steps, are specific to each nucleus [8].

## 5. The Prosomeric Hypothalamic Model

**Figure 2:**
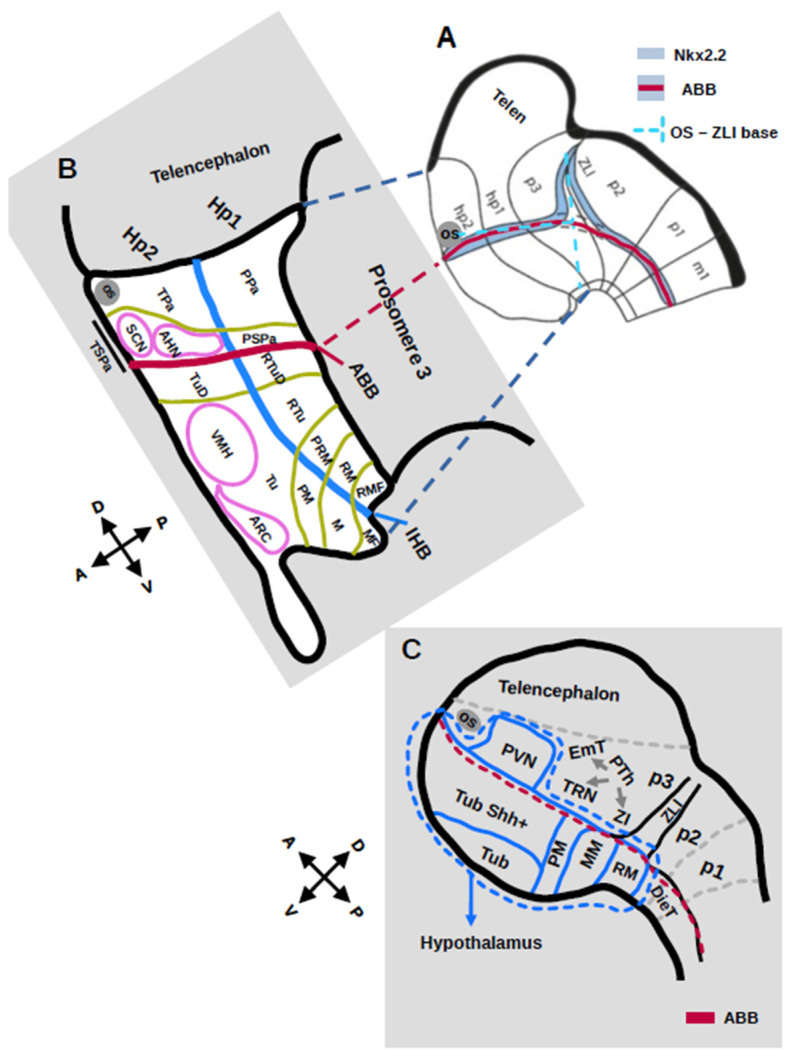
Schematic comparison of the hypothalamic subdivisions in the prosomeric (**A**,**B**) and tripartite model (**C**). Arrows indicate the A-P and D-V axes and show that the tripartite model (**C**) is rotated 90° to the prosomeric model (**A**,**B**). *Nkx2.2* expression, in red, is considered as the Alar/Basal boundary (ABB). In (**A**), an orthogonal angle is also designed at the base of the ZLI (cyan dotted lines) to facilitate identification of the regional position. In (**B**), anterior prosomeres are magnified to show hypothalamic subdivisions. The blue line segments the hypothalamus into two prosomeres, Hp1 (peduncular) and Hp2 (terminal), olive green lines segments prosomeric hypothalamic domains/areas, and pink lines mark examples of conventional nuclei. In (**C**), the blue dotted line delineates the developing hypothalamus, and the small arrows mark the prethalamic (PTh) nuclei. To facilitate the reader, grey dashed lines position potential prosomeric areas (p1–p3) although the tripartite model does not support prosomeric subdivisions. For abbreviations, see Table 1. Adapted from [5,6,59].

According to the prosomeric model, the hypothalamus is part of the anterior forebrain, ventral to the telencephalon and anterior to the diencephalon. Along the A-P axis, the hypothalamus is divided into two parts: posterior, the peduncular hypothalamus (PHy) belonging to hypothalamic prosomere 1 (Hp1) and anterior, the terminal hypothalamus (THy) belonging to hypothalamic prosomere 2 (Hp2, Figure 2A,B). The terminal hypothalamus contains the singular median acroterminal domain (ATD) [5,47]. Along the alar/basal axis, the hypothalamus is divided into the alar peduncular and terminal paraventricular and subparaventricular areas, the basal retrotuberal and tuberal, and basal retromammillary and mammillary areas in the Hp1 and Hp2, respectively (Figure 2B). Each of the alar and basal areas is further divided into zones depending on the position in the area, e.g., dorsal, central/intermediate, ventral or lateral.

The topographical correspondence between the prosomeric domains and conventional nuclei is as follows (Figure 2B):

- In the alar plate, the peduncular paraventricular area (PPa) includes the PVN, the terminal paraventricular area (TPa) includes the periventricular hypothalamic nucleus (PeVN), the peduncular subparaventricular area (PSPa) contains the entopeduncular nucleus (EnN), and the terminal subparaventricular area (TSPa) contains the AHN, part of the SON and lateroanterior nucleus (LAN).
- In the basal plate, the peduncular retrotuberal area (RTu) includes part of the DMH, the posterobasal nucleus (PBN) and part of the LH. The terminal tuberal area (Tu) includes the other part of the DMH, VMH and part of the LH. The Acroterminal domain (ATD) contains the alar lamina terminalis, SON, anterobasal nucleus (ABN), SCN and chiasmatic areas, as well as the ARC, median eminence, and infundibular/neurohypophysial areas. The periretromammillary (PRM) and perimammillary (PM) areas include the dorsal and ventral PMN. The retromammillary area (RM) includes the RMN. The mammillary area (MM) includes the MMN [5,47,57,58,59,60].

These hypothalamic subdivisions are originally defined by the expression of patterning genes [47]. The basal part of the hypothalamus is distinguished from its alar part by *Shh* expression in the ventricular zone and *Nkx2.1* in the mantle layer. *Otp* and *Sim1* are expressed in the alar peduncular dorsal, central, and ventral paraventricular areas and the alar terminal dorsal, central, and ventral paraventricular areas [47,60,61]. *Arx* is expressed in the alar PSPa and the alar TSPa. *Nkx2.1* is expressed in the basal VMH and M and the acroterminal ARC and neurohypophysis. *Nkx2.1* and *Otp* are co-expressed in the basal peduncular PRM, the basal terminal dorsal tuberal area (TuD) and basal terminal PM. *Nkx2.1* and *Dlx5* are expressed in the basal peduncular dorsal and intermediate RTu and the basal terminal intermediate Tu. *Arx*, *Nk2.1* and *Dlx5* are expressed in the basal peduncular ventral RTu and the terminal ventral Tu [47,59]. The hypothalamic floorplate reaches up to the MM and RM, characterized by the epichordal expression of *Shh*, *Ntn1*, *Lmxb1*, *Foxa1*, and *Nr4a2* [47].

Additional gene expression patterns have been analyzed, taking into account the prosomeric model subdivisions. For instance, *Erb4*, *Irx1/3/5*, *Lmo4*, *Mfap4*, *Plagl1*, *Pmch* are expressed in the peduncular hypothalamus (Hp1); *Fgf15*, *Gsc*, *Nkx6.2*, *Otx1*, *Zic1/5* are expressed in the terminal hypothalamus (Hp2); and *Fgf8/10/18*, *Otx2*, *Pomc*, *Rx* and *Six6* are expressed in the ATD which belongs to Hp2 [62].

The authors of the prosomeric model point out that differential gene expression satisfies the assumption that differential gene expression patterns should characterize each of the anterior prosomeres Hp1 and Hp2 [47,62]. However, when examined in detail, several gene expression patterns question the existence of the aforementioned hypothalamic divisions. Examples of inconsistency of gene expression patterns and the claimed intrahypothalamic boundary are found in Table 2.

Neither signaling pathway molecule nor transcription factor nor neuromodulator/neuropeptide expression support a boundary separating the hypothalamus in Hp1 and Hp2 prosomeres according to these studies (Table 2). The division of the paraventricular, subparaventricular and perimammillary areas in a peduncular and terminal domain are unsupportive of an entire dorso-ventral boundary across the hypothalamus, orthogonal to the alar-basal boundary (ABB). Furthermore, HCR analysis of gene expression shows that *Fgf15*, *Six3* and *Zic1/5* expression in the terminal hypothalamus is more medial, that is, in the ventricular zone where progenitors lie, whereas *Otp*, *Sim1*, *Mfap4*, *Lmo4* and *Rgs4* expression is more lateral where cells start to differentiate, when compared to E13.5 sections from the Allen Mouse Brain Atlas [6,63]. scRNAseq analysis of the pooled E11-E14 mouse hypothalamus is unsupportive of hypothalamic peduncular and terminal subdivisions [8]. In contrast, the TPa *Fgf15* and *Six3* are expressed in a single progenitor cluster whereas *Zic1* expression is found in eight clusters. The PPa markers *Mfap4*, *Rgs4*, and *Lmo4* are also variably expressed. Altogether, the scRNAseq clusters suggest that TPa and PPa areas reflect differentiating cells remaining apart from progenitors [6].

**Figure 3:**
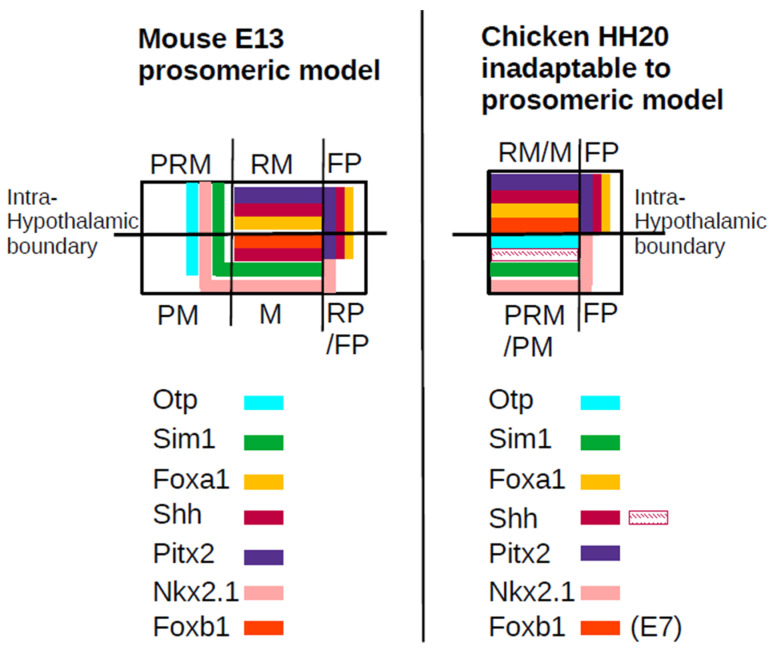
Attempt to transfer gene expression patterns from the mouse hypothalamic prosomeric model to the chicken hypothalamus using colors to code gene expression. The subdivisions claimed by the mouse E13 hypothalamus prosomeric model are unidentifiable in the chick HH20 hypothalamus.

Furthermore, surprisingly, the prosomeric mammillary domain (M) cannot be identified in the chicken hypothalamus [6]. According to the prosomeric model data, the posterior ventral hypothalamus is organized as follows (Figure 3):

A domain expressing *Sim1/Otp* (PRM/PM) and a domain expressing *Nkx2-1/Sim1/Foxb1*, that is, M, a domain expressing *Pitx2/Foxa1* (RM). *Pitx2* expression is found in the RM domain; however, its expression covers the floorplate of both RM and a large part of M domains, and a small *Shh/Foxa1* midline region corresponding to the Hp2 floorplate. The Hp1 prosomere floorplate is defined as extending beyond *Arx*, up to the anterior limit of *Shh/Foxa1* expression (Figure 3).

Unexpectedly, the prosomeric model boundaries cannot be found in the HH20 embryonic chicken brain using the same markers: *Pitx2* is found in a single territory rather than two distinct RM and M domains, *Otp* is expressed in an adjacent anterior single territory rather than distinct PRM and PM domains, and *Foxb1*, at E7, is expressed in a single territory rather than RM and M split fields. In other words, *Nkx2.1/Sim1/Foxa1* and *Otp/Pitx2* boundaries coincide, instead of intersecting orthogonally, so that *Foxa1* and *Otp* directly abut. Therefore, a *Sim1/Nkx2.1* expressing, devoid of *Foxa1/Otp* expression M domain, could not be identified in the HH20 and E7 chicken (Figure 3) [6].

According to the prosomeric model, the fornix characterizes and belongs to Hp1 since it originates in the hippocampus [5]. It branches in relation to the anterior commissure and extends posterior towards its target, the mammillary hypothalamus, where it bifurcates and innervates part of RM and M. However, this is inconsistent with the existence of a hypothalamic boundary since the tract innervates areas in both sides of the boundary. Furthermore, the fornix bundle develops later than the hypothalamic regionalization takes place, at gestational day 16, whereas hypothalamic regionalization is obvious in E12-E13 mice; thus, it is difficult to conceive that it plays a role in prosomere establishment. Finally, although prosomeric Hp1 and Hp2 regionalization is claimed in teleost fish, the fornix tract is not obvious as the hippocampus does not form [64,65].

Finally, the prosomeric model posits a boundary between prethalamus (p3) and hypothalamus (Hp1 and Hp2). In other words, prosomere 3 (p3) contains the prethalamus but the hypothalamus belongs to a more anterior prosomere [5,47]. However, functional experiments are unsupportive of this boundary. As discussed earlier, hypothalamic and prethalamic formation are tightly bound. HypFP cells recruit prethalamic cells to form the hypothalamus [13]. *Nkx2.1* not only promotes and maintains the identity of ventral hypothalamic progenitors but also actively represses prethalamic gene expression [8]. FST from the prethalamus restricts hypothalamic size, and in HH8 chicken, prethalamic and hypothalamic scRNAseq and RNA velocity show a trajectory from prethalamic-like to HypFP clusters [13]. In conclusion, prethalamic and hypothalamic formation share a common territory.

## 6. The Tripartite Hypothalamic Model

**Figure 4:**
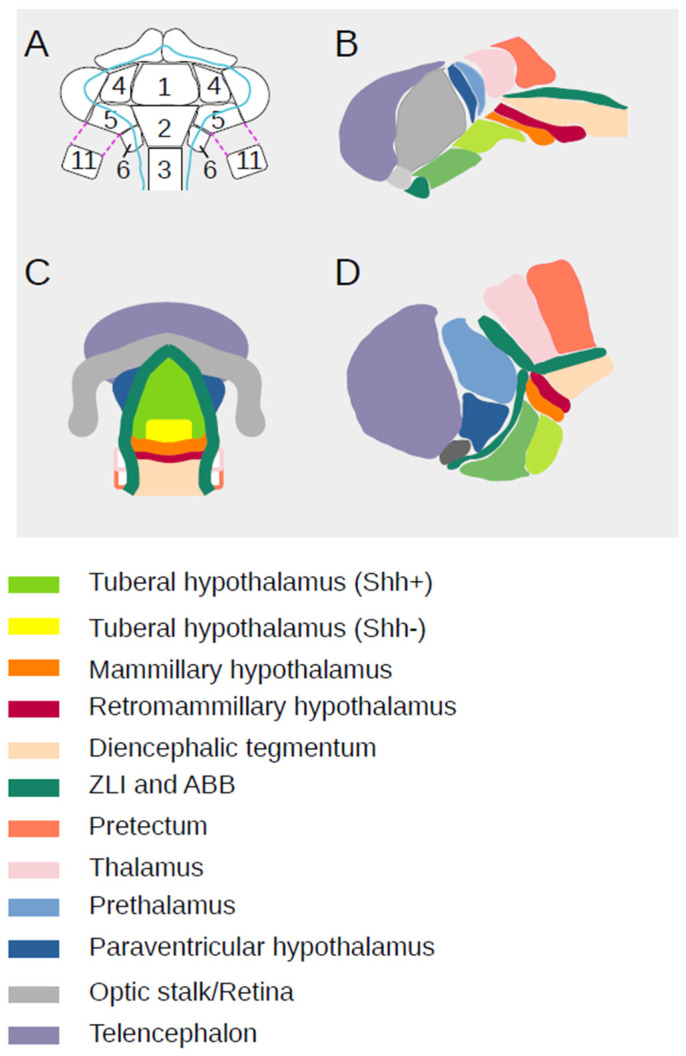
The tripartite hypothalamic model. (**A**,**B**): Cartoons showing a ventral view outline and a mid-sagittal section of the chicken anterior brain at HH10. (**C**,**D**) Cartoons showing a ventral and a lateral view of the HH20 chicken anterior brain. The numbers in (**A**) correspond to the sites of dye injection performed to mark growth lines. Area 11 is localized dorsally to area 5 (pink dotted lines). Only the areas generating the hypothalamic subdivisions are numerically marked according to [6]. In (**B**–**D**), the color code corresponds to the forming hypothalamic and neighboring forebrain areas. Adapted from [6].

The most complete fate map for the anterior neural tube is provided by (Manning et al., 2025) [6]. Chicken embryos are injected with DiI/DiO in the presumptive forebrain territory at HH10 and analyzed after 48 h (HH17-20) (Figure 4). The A-P axis is considered as parallel to the hypothalamic *Nkx2.2* expression domain and the D-V axis as orthogonal to it. Alternatively, because *Nkx2.2* expression is not always available, the A-P axis can also be defined as parallel to the *Shh* expression stripes in the ventral hypothalamus or as a line drawn perpendicular to ZLI from the ZLI base to the optic stalk opening (Figure 2A–C).

The DiI/DiO injection label territories known as growth lines and show how specific areas, by maintaining their relative topology, grow differentially and directionally to generate the chicken forebrain. Key growth patterns are confirmed by genetic clonal analysis using the *Cre-Cytbow* transgenic line [66] and in *Rfp*-electroporated embryos [6]. Dye targeting of HypFP at two ventral midline positions along the anterior ventral midline, namely zones 1 and 2, label the ventral hypothalamus (Figure 4A). Zone 3, further posterior, gives rise to a non-hypothalamic, diencephalic floorplate (Figure 4A–D). Wider midline injections in zones 1–3 form an arrowhead shape in the A-P axis, showing that midline cells are displaced anterior relative to their more lateral counterparts from HH10 to HH20 (Figure 4A–D) [6]. This is in agreement with previous fate mapping at HH10 where tuberal *Six3* and *Fgf10* expressing cells generate anterior hypothalamic cells [41].

Medial zone 4 and 5 injections extend along the A-P axis in the *Shh* expressing hypothalamus. The lateral parts of these lines stretch from the posterior *Shh*-non-expressing but *Foxd1* expressing hypothalamic region, across the alar-basal boundary (ABB), towards the ventral optic stalk and temporal retina. Zone 6 results in a tricorn-shaped domain, ventral to *Nkx2.2* expression and just posterior to the ZLI base (Figure 4A–D). When zones 2 and 6 are targeted simultaneously, an integral-shaped line (∫) pattern extends anteroventral from this tricorn domain through the *Pitx2* expressing domain, towards the tuberal non-*Shh*-expressing hypothalamic midline. Finally, zone 11 is dorsal to zone 5 and expands isometrically to form the PVN hypothalamus and prethalamus. Therefore, fate mapping formally shows a common origin of prethalamic and hypothalamic cells (Figure 4A,D) [6].

This fate mapping analysis shows that at HH20, the hypothalamus comprises the PVN hypothalamus dorsally and the basal hypothalamic area ventrally, containing the cells born from ventral midline and lateral areas. The hypothalamus is segregated anterior and dorsal from the telencephalon by the optic stalk and eye field, dorsal and posterior from the prethalamus, and ventral and posterior by the adjacent diencephalic tegmentum and the beginning of the *Irx3* and *Arx* expressing floorplate. Only the anterior tuberal hypothalamus originates ventral to the telencephalon. The posteroventral hypothalamus arises posterior to the telencephalon and ventral to areas that will give rise to the PVN hypothalamus, prethalamus and thalamus. This is the tripartite hypothalamus model of hypothalamic organization (Figure 2C) [6].

HCR reveals the RMN developmental identity. RMN consists of the *Lfng* non-expressing, *Pitx2*, *Lmx1b* and *Shh* expressing domain, partially overlapping anterior with *Barhl2* expression and lying ventral to the ZLI, posterior to the prethalamus. Anterior to *Pitx2* hypothalamic and floorplate expression lies the domain of *Emx2* expression (MMN), and posterior to it lies the *Arx* expressing floorplate in the diencephalic tegmentum. The *Pitx2* hypothalamic domain is straddled by and partially overlaps with *Sim1*, *EphA7* and *Dbx1* expression, which further reach dorsal and encompass the ZLI. This posterior character is further enhanced by RMN being the anterior domain of *Foxa1* and *Irx1/Irx3* expression immediately posterior to the *Six/Fez* expressing hypothalamus. As a result, the RMN hypothalamus is part of a ventral boundary region associated with lineage restriction. The hypothalamic RMN identity is conserved in the mouse [6].

The tripartite hypothalamic model is further supported by fate conversion. Prospective telencephalon and anterior retina at HH10 express *Foxg1*, prospective prethalamus/PVN hypothalamus expresses *Pax6/Arx*, and optic stalk expresses *Pax2.* When chicken hypothalamus is dorsalized by SHH inhibitors, with or without FST (which restricts hypothalamic size) at a neural tube stage, a small basal territory with *Shh* and decreased *Nkx2.1* expression is retained. Fate mapping confirms that cell movements are not altered. *Pax2* expression is preserved. *Foxg1* is ectopically expressed, but only in part of the anterior-most tuberal hypothalamus, suggesting that this hypothalamic area formation is linked with the telencephalon. More posterior within the tuberal hypothalamus, *Pax6* is ectopically expressed and *Arx* is significantly expanded, showing conversion of the tuberal hypothalamic territory to prethalamus/PVN. Similar results are obtained when HH10 zones 1, 2, 4 and 5 are isolated and cultured in the presence or absence of SHH inhibitors. *Olig2* remains confined to the prethalamus, posterior tuberal and MMN hypothalamus. As a result, the anterior-most tuberal hypothalamus is topologically ventral to the telencephalon, in partial agreement with the prosomere model, but the remainder of the tuberal region is ventral to the PVN hypothalamus and prethalamus and is part of the diencephalon. Fate mapping shows a major association between PVN progenitors and the prethalamus [6].

Furthermore, when PVN, telencephalic and perthalamic cells are isolated in vitro, PVN hypothalamic cells intermingle with prethalamic cells but segregate from telencephalic cells, suggesting that the PVN hypothalamus groups with the prethalamus as part of the diencephalon, posterior to the telencephalon. Basal plate cells taken anterior and posterior to the RM hypothalamus segregate in culture while those from posterior and anterior or ventral positions within the basal hypothalamus intermingle. These results suggest that lineage restriction may operate ventral to the ZLI at the RMN.

Altogether, gene expression, fate mapping, fate conversion and cell intermingling support the tripartite model. According to it, (1) the hypothalamus lies ventral to the telencephalon and to two parts of the diencephalon, the prethalamus and the ZLI; (2) the D-V and A-P axes are rotated 90 degrees, relative to the prosomeric model (Figure 2B,C), so that the tuberal hypothalamus is a ventral structure; (3) the anterior forebrain is non-segmented, i.e., does not have an Hp1-Hp2 boundary; and (4) the retromammillary hypothalamus has a more posterior forebrain identity to the rest of the hypothalamus [6].

## 7. Genetic Mutations and Regulatory Networks Support the Tripartite Model

Additional functional studies provide evidence for the regionalization of the hypothalamus. In particular, genetic mutations and GRN provide robust evidence for the regional boundaries in the forebrain.

The apposition of *Six3* and *Irx3* expression domains corresponds to the zona limitans intrathalamica (ZLI), the boundary-cell population between prethalamus and thalamus. Removal of *Six3* activity in *Six3-/-* mice results in severe forebrain truncations anterior to the ZLI in the alar plate. In *Six3-/-* mice, telencephalic *Foxg1* and retinal/anterior hypothalamus *Rx* expression are absent. *Nkx2.1* expression is absent in the floor of the truncated forebrain and the anterior and tuberal hypothalamus. *Shh* expression in the forebrain basal plate is reduced in length but extended into the rostral end of the truncated forebrain. *Shh* expression is detectable in the ZLI of the *Six3*−/− embryos and *Pax6*, a prethalamus marker in the alar plate, extends to the anterior end of the truncated forebrain. These results contribute to the idea that there is a boundary between the hypothalamus anterior and diencephalic prosomere 3 posterior and that the hypothalamus belongs to an anterior prosomere, distinct from p3 [67].

However, a posterior hypothalamic area persists. *Nkx2.1* is expressed in a small area of the basal plate in the posterior hypothalamus or posterior to it, and *Shh* overlaps with the residual domain of *Nkx2.1* expression [67]. This domain expresses *Irx3*; therefore, a ventral *Shh* expressing domain, at the limits of *Nkx2.1* expression, depends on posteriorizing *Irx3* activity. Within this domain, ventral to the ZLI lies the *Pitx2* expressing domain that corresponds to the RMN. These results suggest that the RMN is the ventral A-P boundary of the ventral forebrain [6].

Both prosomeric and tripartite models agree in the presence of the alar basal hypothalamic boundary along *Nkx2.2* expression [5,6]. *Nkx2.2* is a *Shh*-responsive TF expressed in the dorsal tuberal, ventral prethalamus, and ID. GRN analysis of *Nkx2.2* null mice at E12.5 predicts that *Nkx2.2* directly activates TFs specific to the tuberal hypothalamus, ID and prethalamus and represses RMN-specific TFs. In agreement with this, scRNA-seq of homozygous *Nkx2.2* mutants at E12.5 shows reduced numbers of ID and prethalamus and increased numbers of RMN cells. A mutant-specific cell cluster is observed co-expressing prethalamus and RMN/MMN genes such as *Sp9* and *Meis2*, and *Pitx2* and *Irx5*, respectively. These data infer that *Nkx2.2* expression maintains regional boundaries by promoting prethalamic and tuberal fates while repressing posterior hypothalamic regions, such as the RMN [8].

*Nkx2.1* expression, as discussed earlier, is necessary to promote and maintain mediobasal hypothalamic identity and repress prethalamic fate [9,14]. Therefore, the formations of the prethalamus and hypothalamus are tightly linked.

*Isl1* is expressed in both neurogenic progenitors and postmitotic precursors across a domain extending from the prethalamus, through the ID, and into the tuberal and PMN regions. RNAseq analysis predicts that *Isl1* activates TFs in the prethalamus, ID, tuberal and PMN and represses those in the RMN, MMN, and EmT, an area of the anterior prethalamus. Complementary to this, in *Isl1*-null mice, prethalamus and PMN cell populations are reduced and a mutant-specific cluster co-expressing EmT-like genes (*Tbr1*, *Slc17a6*) and RMN/MMN genes emerges [8]. Therefore, this analysis postulates that the hypothalamus and prethalamus share a common domain/pool of progenitor cells.

GRN predict that *Dlx1/2* activate *Ascl1* and multiple genes specifying prethalamus, ID, and PMN while repressing *Neurog2* and genes driving RMN, MMN and some tuberal cell types. scRNA-seq and scATAC-seq analyses of conditional mutants *Foxd1*-*Cre;Dlx1/2lox/lox* show reduction in *Ascl1* expressing neural progenitors and loss of prethalamus/ID and PMN neural precursors. GABAergic gene expression (e.g.,*Slc32a1*) is also downregulated. Conversely, RMN-like cells expand, with ectopic expression of *Nr4a2* and *Barhl2* in regions typically producing prethalamic neurons. Histological analysis of *Dlx1/2* mutants at E13.5 confirms the reduced prethalamic/ID expression of *Dlx2*, *Dlx5*, *Isl1*, *Arx*, *Hmx2*, *Pax6*, *Meis2*, *Sp8*, *Sp9* and *Gad2* along with a dorsal expansion of the RMN *Foxa1* and *Pitx2* expression. These data show that *Dlx1/2* promotes prethalamic identity and GABAergic differentiation and represses RMN identity [8]. Therefore, the most posterior hypothalamic area, RMN formation, relies on the restriction of prethalamic territory which lies anterior and dorsal to RMN.

*Lhx2* expression maintains optic identity by continuously suppressing alternative fates corresponding to EmT and the antero-dorsal hypothalamus. Expression pattern analysis in tissue and microarrays of E13.5 *Chx10-Cre;Lhx2lox/lox* mice retinae and control littermates show that mutant retinae have substantially reduced expression of *Chx10*, *Foxn4*, *Rax*, and *Rorb*, *Prdm1* and *Crx*. In contrast, genes selectively expressed in the EmT and anterodorsal hypothalamus, such as *Lhx1*, *Lhx5*, *Tbr1*, *Sim1* and *Otp*, are dramatically upregulated so that their expression is greatly expanded and shifted to enter into the telencephalic caudal ganglionic eminence. The posteroventral hypothalamus shows similar patterns of *Shh* and *Nkx2.1* expression in mutant and control brains. In conclusion, *Lhx2* functions to suppress alternative regional fates in the optic vesicle and to pattern the structures surrounding the optic vesicle [68].

In conclusion, a pool of TFs shape the hypothalamus ventral to the telencephalon, optic stalk, prethalamus and ZLI. *Lhx2* activity is required for the telencephalic and anterodorsal hypothalamus. *Nkx2.1* is required for the formation of the mediobasal hypothalamus. Disrupting *Isl1*, *Nkx2.2* or *Dlx1/2* activity results in loss of prethalamic identity and ectopic induction of genes specific to the retromammillary hypothalamus, strong evidence for the interlinked formation of these two areas and absence of a boundary between them.

## 8. Hypothalamic Regionalization in Zebrafish

The prosomeric model has been applied in the zebrafish hypothalamus, but fate mapping is missing. However, in mice, gene expression patterns are analyzed during relatively late hypothalamic development. For example, in Figures 3a and 4, in the study by Schredelseker and colleagues [65], the prosomeric model is taken for given and hypothalamic subdivisions are applied according to it rather than borders of gene expression taken into account to attribute the underlying pattern.

In other words, prosomeric boundaries for M, RM, PM, and PRM do not coincide with distinct gene expression limits. In addition, the floorplate cannot be faithfully distinguished at 48 hpf in the hypothalamus nor in the posterior diencephalon. Nevertheless, all the genes expressed in the boundary area of the tripartite hypothalamic development model are available in zebrafish, including *Pitx2*, *Emx2*, *Arx*, *Wnt8b*, *Lmx1b*, *Barhl2* and *Irx3* [27,69,70,71,72,73,74,75,76], and their expression profile could be analyzed with respect to hypothalamic/floorplate development at the right developmental period. For instance, *Arx* expression in the posterior diencephalic floorplate is shown in Figure 4J in [74] at 40 hpf, suggesting that a similar boundary domain is also formed at about 30–45 hpf.

## 9. Conclusions

Through thorough fate mapping, gene expression pattern, scRNAseq analysis and functional assays, Manning and colleagues [6] show that the major part of the hypothalamus develops ventral to the PVN hypothalamus, prethalamus and ZLI. The retromammillary region characterized by *Lnfg* non-expressing and *Pitx2*, *Irx3*, *Foxa1* expressing cells has a posterior identity and likely resides in a boundary region between the hypothalamus and diencephalic tegmentum, ventral to ZLI another diencephalic thalamus/prethalamus boundary. Genetic and GRN analysis support the tripartite model. The ZLI and RM diencephalic continuum is likely to be conserved in all vertebrate species.

## Figures and Tables

**Table 1:** Abbreviations.

**ABB**	alar-basal boundary
**ABN**	anterobasal nucleus
**AHN**	anterior hypothalamic nucleus
**A-P**	anteroposterior
**ARC**	arcuate nucleus
**ATD**	acroterminal domain
**DMH**	dorsomedial hypothalamic nucleus
**D-V**	dorsoventral
**EmT**	thalamic eminentia
**EnN**	entopeduncular nucleus
**FP**	floorplate
**GRN**	gene regulatory networks
**HH1**	Hamburger–Hamilton stage 1
**HP1**	hypothalamic prosomere 1
**HP2**	hypothalamic prosomere 2
**HypFP**	hypothalamic floorplate-like cells
**ID**	intrahypothalamic diagonal
**LAN**	lateroanterior nucleus
**LH**	lateral hypothalamic area
**M/MM**	mammillary area
**MMN**	mammillary hypothalamic nucleus
**os**	optic stalk
**p1, p2, p3**	Prosomere 1, 2, 3
**PBN**	posterobasal nucleus
**PeVN**	periventricular hypothalamic nucleus
**PHN**	posterior hypothalamic nucleus
**PHy**	peduncular hypothalamus
**PM**	perimammillary area
**PMN**	premammillary hypothalamic nucleus
**Ppa**	peduncular paraventricular area
**PSPa**	peduncular subparaventricular area
**Pth**	prethalamus
**PVN**	paraventricular hypothalamic nucleus
**RM**	retromammillary area
**RMN**	retromammillary hypothalamic nucleus
**Rtu**	retrotuberal area
**SCN**	suprachiasmatic nucleus
**scRNAseq**	single cell RNA sequencing
**SON**	supraoptic nucleus
**THy**	terminal hypothalamus
**Tpa**	terminal paraventricular area
**TRN**	thalamic reticular nucleus (prethalamus)
**TSPa**	terminal subparaventricular area
**TT**	tuberomammillary terminal
**Tu**	tuberal domain
**TuD**	dorsal tuberal domain
**TuN**	tuberal nucleus (migrated from VMH)
**VMH**	ventromedial hypothalamic nucleus
**ZI**	zona incerta

**Table 2:** Examples of gene expression domains unsupportive of the hypothalamic prosomeres 1 and 2.

Gene	Expression Domain	Supporting Concept	Experimental Evidence
Otp	Prethalamus and Hypothalamus (E13.5)	Continuity p3 and hypothalamus	Figure 3E in [62]
Otp	Peduncular Ppa and Terminal Tpa	Absence of intrahypothalamic boundary	Figure 3E in [62]
			Figures 2A and 3L,O in [59]
Otp	Periretromammillary PRM and	Absence of intrahypothalamic boundary	Figure 3E in [62]
	Perimammillary PM areas		
Dlx5	Prethalamus and Hypothalamus (E10.5-13.5)	Continuity p3 and hypothalamus	Figure 2A in [60]
			Figures 3I and 4F,H in [59]
Sim1	Peduncular Ppa and Terminal Tpa (E13.5)	Absence of intrahypothalamic boundary	Figure 2D in [59]
Plagl1	Peduncular DM-P, PRM, Terminal Hypothalamus	Absence of intrahypothalamic boundary	Figures 3R and 5A–D in [62]
	(E15.5-P4)		
Rgs4	Nuclei throughout Hypothalamus (E13.5-P4)	Absence of intrahypothalamic boundary	Figure 3I–L in [62]
		(If the ventral premammillary nucleus migrates from RM area, the idea of boundary collapses)	
Zic5, Zic1	Peduncular Ppa and Terminal Tpa (E13.5-P4)	Absence of intrahypothalamic boundary	Figure 3A–D,F (compare Figure 3A,B,F ventricle with Figure 3C,D lateral) in [62]
		
Meis2	Peduncular PSPa and Terminal TSPa (E13.5-18.5)	Absence of intrahypothalamic boundary	Figure 4A,B in [62]
TH immuno-	Peduncular Ppa and Terminal Tpa (adolescence)	Absence of intrahypothalamic boundary	Figures 2A,B and 5A–D in [57]
reactivity	PRM,RM, PM, M (adolescence)	Absence of intrahypothalamic boundary	Figures 7 and 8 in [57]

## Data Availability

Data is contained within the article. The original contributions presented in this study are included in the article. Further inquiries can be directed to the corresponding author.
